# Treatment outcomes of paediatric chest wall Ewing’s sarcoma; report from tertiary care cancer center

**DOI:** 10.3332/ecancer.2026.2117

**Published:** 2026-05-01

**Authors:** Rabia Wali, Sadia Anjum, Sumera Abdul Kareem, Najma Shaheen

**Affiliations:** 1 Department of Paediatric Oncology, Shaukat Khanum Memorial Cancer Hospital and Research Centre, Lahore 54000, Pakistan; 2 Department of Radiation Oncology, Shaukat Khanum Memorial Cancer Hospital and Research Centre, Lahore 54000, Pakistan; 3 Department of Paediatric Hematology and Oncology, University of Child Health Sciences, Lahore 54600, Pakistan; a https://orcid.org/0000-0002-8252-3977

**Keywords:** Ewing sarcoma, chest wall, paediatric oncology, chemotherapy, survival analysis, surgical margins

## Abstract

**Introduction:**

Chest wall Ewing sarcoma (EWS) is a common paediatric malignant tumour affecting the chest wall, accounting for about 10% of all EWS patients. Treatment typically involves intense chemotherapy, complete surgical resection when possible and/or radiation. The EWS of the chest wall has a reported 5-year survival ranging between 40% and 60% in most studies. The following study evaluated the effect of different treatment modalities on survival outcomes in paediatric patients.

**Methodology:**

This is a single-center retrospective cohort study that included 46 patients diagnosed with chest wall EWS between January 2011 and December 2023 at Shaukat Khanum Memorial Cancer Hospital and Research Center. Approval was obtained from the Institutional Review Board for this study. The 5-year follow up period and outcomes were analysed using the SPSS.

**Results:**

The cohort included 46 patients (56% female, 44% male) with a median age of 10 years. Non-metastatic disease was observed in 94% of the patients. The compressed vincristine, doxorubicin, cyclophosphamide, ifosfamide and etoposide chemotherapy regimen showed better outcomes, with a 48% survival rate compared with 38% for vincristine, doxorubicin, actinomycin, ifosfamide and etoposide. Negative surgical margins were associated with a 56% survival rate. 5-year overall survival (OS) of 42.5% and event-free survival (EFS) of 39%. Both the OS and EFS were better in patients who had surgery alone as local control (LC).

**Conclusion:**

Outcomes in patients who had surgery as LC did better in this study. Early recognition and referral may help in reducing the tumour burden so that good LC with surgical R-zero resection is possible.

## Introduction

Ewing sarcoma (EWS) is an aggressive malignant tumour that mainly affects children and adolescents, with the chest wall being one of the common sites where it can develop [1]. This tumour type arises from primitive neuroectodermal tissue and is characterised by translocation involving the EWSR1 gene, which results in the production of a fusion protein that promotes tumour growth [2]. EWS accounts for about 10% of all paediatric chest wall cancers [3]. Managing paediatric EWS of the chest wall presents significant challenges due to the tumour’s location and its potential for metastasis [4].

Chemotherapy followed by surgical resection and radiotherapy (RT) is the primary treatment for EWS, aiming to achieve the best optimal tumour response while minimising complications and preserving function [5]. Surgical approaches often involve complex multidisciplinary planning, including thoracic, spinal surgery and orthopedic oncology, to ensure complete excision of the tumour while maintaining chest wall integrity [6]. Chemotherapy plays a critical role in managing systemic diseases, improving survival rates and reducing the risk of local recurrences. Despite advancements in treatment strategies, the reported 5-year overall survival (OS) rate for patients with chest wall EWS remains between 40% and 60% [7]. Managing EWS is further complicated by its aggressive clinical behaviour, rapid metastatic potential and high recurrence rate [8]. Tumours with associated malignant pleural effusion and nodules can add to the complexities in achieving local control (LC). Since chest wall involvement is less common, clinical outcome data are limited. Given the long-term side effects of treatment, including cardiac and pulmonary complications, growth disturbances and secondary malignancies associated with chemotherapy or radiation, long-term follow-up is essential to monitor for recurrence and late complications [9]. In recent years, advances in imaging techniques, surgical approaches and chemotherapy protocols have significantly improved the outcomes of EWS [10].

Given the rarity of this condition, this study aimed to evaluate the clinical outcomes of paediatric patients diagnosed with EWS of the chest wall who were treated with chemotherapy, surgical resection where possible and RT in the setting of a low-income country. Outcomes based on primary treatment modalities, including chemotherapy regimens and LC, along with tumour size, were examined.

## Methodology

This single-center retrospective cohort study was conducted at the Shaukat Khanum Memorial Cancer Hospital and Research Centre, Lahore, Pakistan. The study included all paediatric patients under 18 years of age diagnosed with EWS of the chest wall between January 2011 and December 2022. Newly diagnosed patients with chest wall Ewing confirmed on histology were included in the analysis. Data were gathered from the hospital’s electronic medical records, including the following parameters: (1) demographics: age, sex and socioeconomic status, (2) tumour characteristics: tumour size, location, histopathology and staging, (3) treatment details: chemotherapy regimens, surgical resection and RT and (4) outcomes: OS, event-free survival (EFS). Approval was obtained from the Institutional Review Board (EX-16-10-19-A2).

From March 2011 to February 2017, vincristine, doxorubicin, actinomycin, Ifosfamide and etoposide (VIDE/VAI) regimen was administered at a standard 3-week interval. In April 2017, the treatment regimen was shifted to a biweekly, intensified chemotherapy protocol – the vincristine, doxorubicin, cyclophosphamide, Ifosfamide and etoposide (VDC/IE) protocol – which showed improved survival outcomes [11]. The switch was done as delivered in an outpatient setting, reducing inpatient bed use and wait times. Three cycles of each VDC and IE were given 2 weeks apart. Reassessment scans were done after five cycles of neoadjuvant chemo for both protocols. LC was achieved through surgery, RT or both, done after six cycles of chemotherapy. A chest computed tomography (CT scan) was performed at baseline and at re-assessment. A bone scan and bilateral bone marrow aspiration biopsy were done at baseline to identify metastatic disease. RT was given to patients with positive surgical margins and for whom surgery was not possible. The radiation dose ranged from 40 to 55.8 Gys, depending on surgical margins, resection completeness and resectability, for both chemo-regimens. The final follow up date for this study was 31st December 2023, and 5-year outcomes were performed. OS was defined as the time from diagnosis to last follow-up or death (due to disease or other causes), whichever came first. EFS is the time between a diagnosis and recurrence, progression or death. An Excel was designed to collect the variables and survival probabilities were performed by using Kaplan–Meier survival analysis for over-all outcomes, between different treatment modalities and two chemo-regimens. The rest of the analysis across groups is univariate and bivariate descriptive statistics. Statistical analyses were conducted using SPSS version 29.0, IBM Corp. (USA).

## Results

A total of 46 patients with EWS of the chest wall were included in this study. The cohort was composed 56% females and 44% males and 56% were older than 10 years. The age range was from 0.7 to 16 years, with a median age of 10 years. Most tumours originated from the ribs (78%), followed by the thoracic spine (15%) and clavicle/shoulder (7%). Fluorescence in situ hybridization for EWSR1-FLI-1 was performed in 65% of patients after it was introduced by the pathology department in our institution in 2014. Most patients (94%) had non-metastatic disease. At the end of treatment, 39% of cases were in complete remission. At the end of the analysis, 45% are alive, while 55% had died. Disease progression was the main cause of death in 83% of patients, mainly occurring after local treatment and just before the completion of adjuvant chemotherapy.

Treatment-related deaths were observed in four patients (17%) caused by bacteremia-related sepsis and cardiogenic shock. One patient abandoned treatment and later died due to disease progression. Three patients developed myocardial dysfunction – one during adjuvant chemotherapy and two more developed it 2 years after treatment. Additionally, one patient developed secondary acute lymphoblastic leukaemia 2 years post-treatment 2 year and died from sepsis.

Table 1 shows the patient characteristics for both groups, VIDE/VAI and VDC/IE, while Table 2 shows outcomes for the two chemotherapy protocols. Among the 33 patients treated with VDC/IE, 48% survived at the end of the study and disease progression was noted in 39%. In contrast, only 38% of the 13 patients who received VIDE/VAI survived, with disease progression noted in 62%. However, the difference between the two groups is not statistically significant (*p*-value = 0.72). Outcomes based on the tumour size were also analysed: of the 29 patients who underwent surgery, 19 (66%) had tumour size larger than 8 cm and 9 (47%) out of them are alive and in remission. The remaining died due to disease progression. For patients with tumour size smaller than 8 cm, 50% are alive while the rest died due to relapse and treatment-related toxicity (Table 3).

The outcomes of the 29 patients who underwent surgery (*n* = 5) and surgery plus RT (*n* = 24) were analysed based on surgical margin status. Of the remaining patients (*n* = 16), some had resection from outside hospitals or were considered unresectable by the surgeon; RT was given to 13 of these 16. Two patients had disease progression before RT could be started, and one patient’s family refused treatment. No second-look surgery was performed on patients who underwent outside hospital resection. Sixteen patients (55%) had negative surgical margins with over 90% necrosis, while five (17%) patients had positive margins. The remaining eight patients (27.5%) could not have their surgical margins evaluated because their specimens were fragmented or considered inadequate, often due to being sent from outside hospitals. This included patients with chest Ewing’s with spinal involvement, where margins could not be assessed. Patients with negative margins showed better survival outcomes, with 56% surviving at the end of the study. Conversely, only 40% of the patients with positive margins survived. All the deaths in this group were caused by to local progression or metastatic disease, except for three patients who died from toxicity. Table 3 shows outcomes of 29 patients who had surgery with tumour size, margins and age.

RT alone as local treatment was given to 13(28%) patients because the chest tumour could not be surgically removed. Of these, seven (54%) are alive and in remission, while the remaining six (46%) died either from local progression or after developing metastatic disease at the end of treatment or on follow up.

**Figure 1.**
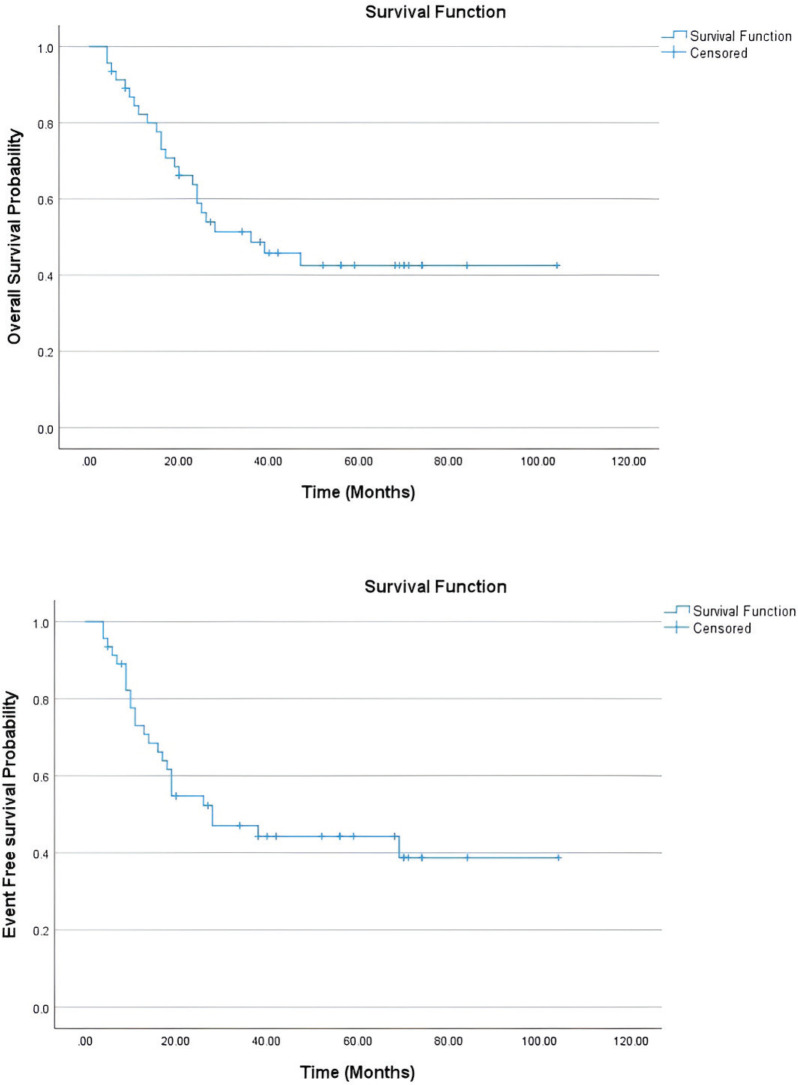
5-year OS and EFS in chest wall EWS.

**Figure 2.**
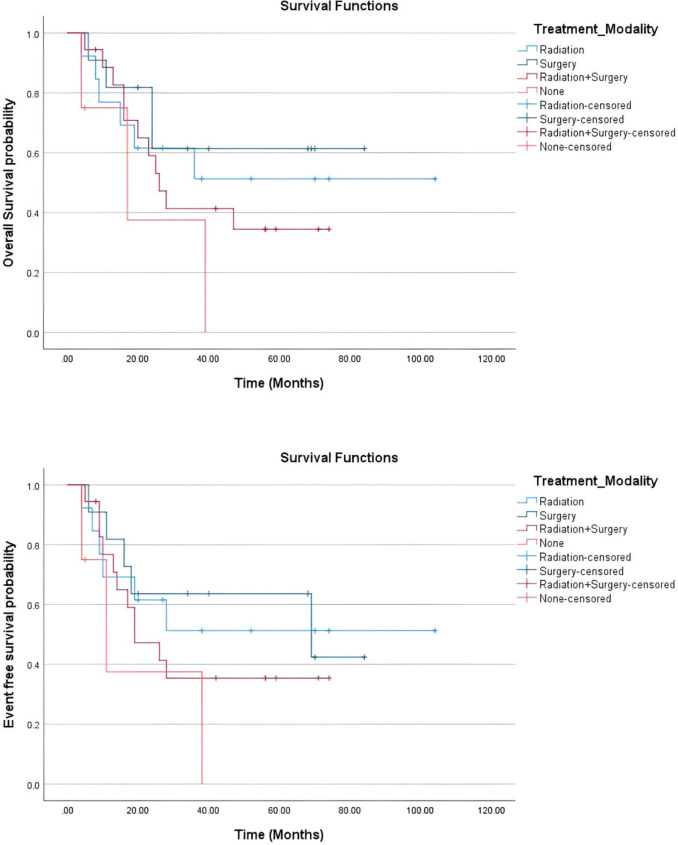
5-year OS and EFS in patients treated with differential LC.

The OS and EFS were analysed using Kaplan–Meier survival curves (Figure 1). The 5-year OS rate is 42.5%, and the 5-year EFS is 39%. OS for patients who had surgery alone for LC was 62%, compared to 52% with RT and 35% for with both surgery and radiation. Similarly, the EFS was better in those patients who had surgery alone, 65%, versus those who received RT alone, 52% and 35% in those with both surgery and RT as LC (Figure 2).

## Discussion

Chest wall EWS is a rare and aggressive tumour that requires multimodal treatment, including surgery and RT, to improve patient outcomes [12]. It often metastasizes and has a higher risk of local recurrence. Reported outcomes of chest wall EWS in the literature vary widely, with 5-year LC, EFS and OS from72% to 97%, 34% to 74% and 34% to 81.6%, respectively [13]. This study presents outcomes of 46 patients with chest wall EWS treated with two chemotherapy regimens, surgery and RT in a lower-middle-income country. Our institution is one of the largest tertiary care cancer center, and we report our experience over the past 12 years.

The peak incidence for EWS happens between 13 and 16 years old, with more male affected. In our study, 56% of patients were aged 10–18 years, with a nearly equal male-to-female ratio of 1:1.2 [14]. In 2017, the treatment protocol was switched to compressed cycles using the Children’s Oncology Group VDC/IE chemo regimen, replacing the Euro-Ewing protocol VIDE/VAI. This change aimed to improve survival outcomes, reduce hospital stays, and prevent delays in chemotherapy administration due to the long waiting list. A feasibility audit was performed to evaluate the outpatient administration of VDC/IE, it was found to be safe. We noted less toxicity and febrile neutropenic complications with this combination and patient tolerated it well [15].

The comparison of outcomes between VDC/IE and VIDE/VAI chemotherapy regimens revealed notable differences in survival. Patients treated with VDC/IE demonstrated higher survival rates (48% alive) than those treated with VIDE/VAI (38% alive), but this difference was not statistically not significant (*p*-value = 0.72). Because this study is descriptive and therefore underpowered to provide overinterpretation.

Disease progression reached 100% in the VIDE/VAI group, likely due to a delay in starting this regimen on time due to a long waiting admission list and delays in count recovery. Patients tolerated VDC/IE regimen and it was changed to be given in the outpatient setting to prevent no delays.

Ewing’s sarcoma is chemo-radio sensitive tumour, so neo-adjuvant chemotherapy can help reduce the morbidity associated with of surgery, especially in the chest wall. If R zero (RO) resection is achieved, radiation can be avoided. However, removing the soft tissue chest wall mass along with the involved ribs to obtain negative margins is not feasible with large tumour size. In our study, we observed a 56% survival among patients with negative surgical margins. Reported outcomes in the literature range from 60% to 90% [16]. The lower survival rate in our cohort could be due to tumour size larger than 8 cm time, late diagnosis and in five cases, the margin status was unknown because surgeries were performed elsewhere, so no details were available. Shamberger *et al* [22] found an increase in the adverse outcomes for tumours larger than 8 cm and in older patients [14, 17]. This is also reflected in our analysis (Table 3).

Our results showed an overall 5-year survival rate (OS) of 42.5%, with a median survival time of 36 months. The EFS was 39%. In a study by Bedetti, OS was 91% and EFS 65% at 5 years. In a review of 104 patients, Laskar *et al* [25] found an OS of 54% and an EFS of 36%. Seitz *et al* [18] evaluated patients with intrathoracic and chest wall sarcomas and found that the EFS of those with chest wall sarcomas was 62%, compared to 38% for those with intrathoracic sarcomas (*p* = 0.008). Our outcomes are slightly lower than those s reported for OS but comparable to some studies for EFS. This could be due to better salvage options in relapse, as most of the data are reported from high-income countries. Challenges such as increase in tumour size, delayed presentation and less experienced surgical expertise for local resection, which are common for low-income countries, may also negatively impact outcomes [18–20].

OS in patients who underwent surgery alone as LC was better than that of those who received RT or had both surgery and radiation. Similarly, the EFS was higher in patients who had surgery alone than in those who received RT alone or had both surgery and RT as LC.

Complete resection is essential, and studies have shown that patients with non-metastatic disease who undergo complete surgical resection and chemotherapy, with or without RT, experience favorable outcomes and long-term survival [21]. Neo adjuvant chemotherapy helps achieve complete tumour resection along with negative margins, as Ewing’s is very chemo sensitive. Denbo *et al* [21] reported that among 20 patients who had surgical resection, the estimated 15-year survival was 90%. Shamberger *et al* [22] reported an estimated 5-year survival of 61% for patients with nonmetastatic chest wall ES.

The impact of RT on survival is unclear, as no randomised trials have directly compared local treatment (surgery plus RT) with surgery alone. Indelicato *et al*. [9] reviewed the 40 years of experience at the University of Florida treating ES; LC was not statistically significantly different between patients treated with RT alone (61%) and those treated with surgery and RT (75%). Denbo *et al* [21] reported that the 7- year EFS was 63% for the radiation group and 46% in the no radiation group (*p* = 0.13). In this study, the group that did not received RT had larger tumours. Sirvent reported that the local failure rate was not significantly different between complete or incomplete surgery (21%) and between with surgery and RT (22%) [14]. Patients who did not receive RT had small tumours at diagnosis, complete resections and a good response to chemotherapy. In our study, patients who received RT were those for whom surgery was not possible, but did better than those who had surgery plus RT (52% versus 35%). The CESS-81 trial also found no benefit from adding RT, but these findings should be interpreted with caution due to differences in size and localisation by various group. RT is still needed in patients having large and incompletely resected pelvic, paravertebral tumours [23, 24].

Long-term follow-up is crucial for identifying late complications, monitoring recurrence and managing treatment-related toxicities, such as cardiotoxicity and secondary malignancies [2]. In our study, three patients developed myocardial dysfunction during disease progression and one patient had secondary acute lymphoblastic leukaemia 1 year after treatment and died from sepsis.

One of the limitations of this study was its retrospective, single-center design, small sample size, reliance on descriptive statistics and the omission of pleural involvement. Studies show that pleural involvement or effusion as an important prognostic factor and associated with worse survival outcomes, similar to those reported in metastatic Ewing’s Sarcoma [14].

Additionally, our center accepts patients from all across the country and even from across the border which can cause delays in presentation. Patients often have locally advance disease, which effects their treatment response, options for LC and ultimately their outcomes, even though they are treated according to standard of care protocols.

## Conclusion

This study reports the outcomes of chest wall EWS from a resource-limited country. Patients who underwent surgery for LC showed better results. Early recognition and referral may help in reducing the tumour burden, allowing for effective LC with surgical RO resection, which can prevent long-term effects of RT. Access to a multidisciplinary team and close collaboration at the national level are essential to improve outcomes through the development of national treatment guidelines.

## List of abbreviations

EFS, event-free survival; EWS, Ewing sarcoma; LC, local control; OS, overall survival; RT, radiation; VDC/IE, vincristine, doxorubicin, cyclophosphamide, ifosfamide and etoposide; VIDE/VAI, vincristine, doxorubicin, actinomycin, Ifosfamide and etoposide.

## Conflicts of interest

The authors declared no conflicts of interest during study and conduction and submission.

## Funding

This study received no funding.

## Ethical statement

This study was approved by the Institutional Review Board of the Shaukat Khanum Memorial Cancer Hospital & Research Centre (SKMCH), Lahore, Pakistan (Dated: July 2024).

## Patient consent

No consent needed as retrospective data.

## Author contributions

Conception and design: Najma Shaheen, Sadia Anjum, Sumera. A. Karim. Collection and assembly of data: all authors. Data analysis and interpretation: Rabia Wali, Sadia Anjum. Manuscript writing: Rabia Wali, Najma Shaheen, Sadia Anjum. Final approval of manuscript: All authors. Accountable for all aspects of the work: All authors.

## Data availability

Available on request.

## Figures and Tables

**Table 1.** Patient characteristics.

Characteristics	VIDE/VAI N = 13 (28%)	VDC/IE N = 33 (72%)
Age		
a. >10 years	8	18
b. <10 years	5	15
Site		
a. Ribs	9	22
b. Thoracic spine	0	9
c. Clavicle/shoulder	4	2
LC		
a. Surgery	1	4
b. Surgery + RT	5	19
c. RT only	4	9
d. None	3 (died)	1 (died)
Disease at EOT		
a. Complete remission	5	13
b. Residual disease	0	5
c. Stable disease	2	6
d. Primary site progression	5	2
e. Metastatic relapse	0	7
f. Abandoned treatment	1	0

**Table 2.** Outcomes comparison between VDC/IE and VIDE/VAI chemo regimens (*N* = 46).

Protocol outcomes	VDC/IE protocol N = 33 (72%)	VIDE/VAI N = 13 (28%)
Alive	16 (48%)	5 (38%)
Death	17 (52%)	8 (62%)
Causes of death		
Sepsis	4 (24%)	--
Disease progression	13 (76%)	8 (100%)

**Table 3.** Outcomes comparison with surgical margins, tumour sizes and age (*N* = 29).

Outcomes variables		Alive	Dead
Surgical margins *N* = 29	Negative surgical margins- 16 (55%)	9 (56%)	7 (44%)
	Positive surgical margins- 5 (17%)	2 (40%)	3 (60%)
	Unknown- 8 (28%)	3 (38%)	5 (62%)
Tumour size at diagnosis *N* = 29	>8 cm- 19 (66%)	9 (47%)	10 (53%)
	<8 cm (33%)	5 (50%)	5 (50%)
Age at diagnosis *N* = 29	>10 years -17 (59%)	8 (47%)	9 (53%)
	<10 years -12 (41%)	6 (50%)	6 (50%)
